# Detection of New Quorum Sensing *N*-Acyl Homoserine Lactones From *Aeromonas veronii*

**DOI:** 10.3389/fmicb.2018.01712

**Published:** 2018-07-31

**Authors:** Meng Gui, Lei Liu, Ruiyun Wu, Jingrong Hu, Shun Wang, Pinglan Li

**Affiliations:** ^1^Beijing Advanced Innovation Center for Food Nutrition and Human Health, College of Food Science and Nutritional Engineering, China Agricultural University, Beijing, China; ^2^Beijing Fisheries Research Institute, Beijing, China; ^3^Beijing Laboratory for Food Quality and Safety, China Agricultural University, Beijing, China; ^4^School of Life Sciences, Inner Mongolia University, Hohhot, China

**Keywords:** *Aeromonas veronii*, specific spoilage organism, sturgeon, acyl homoserine lactone, quorum sensing

## Abstract

Sturgeon is an important fresh water-culture fish in China. A problem with sturgeon is its high susceptibility to spoilage. Food spoilage is reported to be regulated by quorum sensing (QS). To identify the QS signals acetylated homoserine lactones (AHLs) in sturgeon and test whether QS plays a role in the spoilage of sturgeon, we investigated the specific spoilage organisms (SSOs) in vacuum packaged sturgeon stored at 4°C and the production of AHLs by sturgeon SSOs. 16S rDNA sequencing and spoilage capabilities analysis revealed that *Aeromonas veronii* LP-11, *Citrobacter freundii* LPJ-2, and *Raoultella ornithinolytica* LPC-3 were the SSOs in sturgeon. Among the three SSOs, only *A. veronii* LP-11 induced the QS biosensors *Agrobacterium tumefaciens* KYC55 and *Chromobacterium violaceum* CV026, suggesting that it produced AHLs. Analysis by thin layer chromatography, high-performance liquid chromatography-triple quadrupole tandem mass spectrometry, and high-performance liquid chromatography quadrupole time-of-flight mass spectrometry (HPLC/qTOF-MS) identified that the AHLs produced by *A. veronii* were C6-SHL, C8-HSL, 3-oxo-C8-HSL, and 3-OH-C8-HSL. Our study revealed that QS system was probably involved in the regulation of sturgeon spoilage and for the first time reported the production of C8-HSL and 3-OH-C8-HSL by genus *Aeromonas*. As only HPLC/qTOF-MS effectively and accurately identified all the four AHLs produced by *A. veronii* LP-11, this study also showed that HPLC/qTOF-MS was the most efficient method for rapid analysis of AHLs in complex microbial sample. The study provides new insight into the microbiology of sturgeon spoilage which may be helpful for better sturgeon preservation.

## Introduction

Sturgeon is economically one of the most important fresh water-cultured fish in China, of which the production in China takes up to 80% of the total production in the world. One problem with sturgeon is its high susceptibility to spoilage due to the high water content and nutrients. For this reason, refrigeration and vacuum packing are commonly used for the storage and transportation of sturgeon products. Growth of microorganism is the major cause of fish spoilage, which leads to the production of biogenic amines, organic acids, sulfides, alcohols, aldehydes, ketones, and unpleasant off-flavors (Gram and Dalgaard, 2002). The microflora that produces undesirable metabolites and are responsible for food spoilage are called specific spoilage organism (SSO; Gram et al., 2002).

Quorum sensing (QS) is a widespread process in bacteria that employs autoinducing chemical signals to coordinate diverse activities such as bioluminescence, biofilm formation, and exoenzyme secretion (Schuster et al., 2013). QS affects the microflora ecosystem in food, which may finally impacts the sensory quality and safety of food (Rul and Monnet, 2015). Thus, it is likely that QS plays a role in the spoilage of aquatic products. In fact, it has been reported that the secretion of a number of microbial extracellular enzymes, including lipases, nuclease, and protease, is regulated by QS (Skandamis and Nychas, 2012). In addition, QS signals have been detected in a variety of different spoiled commercial aquatic products, such as cold-smoked fish fillets, packed or refrigerated fish, and shrimp (Christensen et al., 2003; Flodgaard et al., 2005; Bai and Rai, 2011; Zhu et al., 2015, 2016). Acetylated homoserine lactones (AHLs) are utilized by a diverse range of Gram-negative bacteria as QS signals. *N*-(3-oxo-hexanoyl)-L-homoserine lactone (3-oxo-C6-HSL) which belongs to AHLs has been determined to be a QS signal in *Serratia proteamaculans* B5a isolated from spoiled cold-smoked salmon to modulate proteolytic activity of the strain, showing the involvement of QS in spoilage of fish (Christensen et al., 2003). AHLs are also detected in *Shewanella putrefaciens*, the SSO of refrigerated shrimp (Zhu et al., 2015). Taken together, these reports demonstrate that AHL-based QS system in Gram-negative bacteria is involved in the spoilage of some aquatic products. Using AHLs, lactonase which degrades AHLs could inhibit the spoilage progress of vacuum-packaged sturgeon stored at 4°C, indicating that QS signals AHLs are implicated in sturgeon spoilage (Gui et al., 2017). However, the mechanism of QS in sturgeon spoilage and their precise role in spoilage is still not clear.

To understand the role of QS in food spoilage, it is necessary to identify the QS signal molecules. As QS can be activated by very low level of AHLs for some strains (Mengsheng et al., 2005), the sensitivity of the analysis method should be very high to detect trace amounts of AHLs produced by the strain. AHL-sensitive bioassay is a convenient method to detect AHLs. Some high-sensitive AHL biosensors, such as *Chromobacterium violaceum*, *Agrobacterium tumefaciens* (TraR), and *Pseudomonas aeruginosa* (LasR), have been developed (Gould et al., 2006). However, there is an inherent detection bias of this method because the analysis relies on the specificity of the reporter strain (Steindler and Venturi, 2007). For this reason, mass spectrometry (MS) is increasingly being applied for the detection and identification of QS signals. MS is currently the most versatile method for rapid analysis of AHLs in complex microbial samples (Cataldi et al., 2013). With the development of MS technology, high-resolution mass spectrometry (HRMS) is becoming feasible and necessary for confident and contemporary QS analysis for it providing highly selective chemical information, such as accurate mass, isotope distribution, and characteristic fragmentation. So far, quadrupole time-of-flight (qTOF) MS as a representative HRMS has been successfully used to detect AHLs in *Pseudomonas putida* IsoF cell culture supernatant (Cataldi et al., 2013).

This objective of this study is to identify the AHLs in SSOs of sturgeon and test whether QS plays a role in the spoilage of sturgeon. For this purpose, we first identified the SSOs of vacuum packaged sturgeon stored at 4°C and then examined the presence of AHLs in SSO culture by different methods.

## Materials and Methods

### Strains and Culture Conditions

*Agrobacterium tumefaciens* KYC55 (pJZ372)(pJZ384)(pJZ410) (Zhu et al., 2003) and *Chromobacterium violaceu*m CV026 (McClean et al., 1997) were kindly offered by Zhigang Zhou (Chinese Academy of Agricultural Sciences, Beijing, China) and used for the detection of AHLs as previous described (Ravn et al., 2001) *A. tumefaciens* KYC55 was grown in LB medium supplemented with 1 μg/mL tetracycline,100 μg/mL tetracycline, and 100 μg/mL spectinomycin. *C. violaceum* CV026 was gown in LB medium supplemented with 20 μg/mL kanamycin. *N*-butyryl-L-homoserine lactone (C4-HSL), *N*-hexanoyl-L-homoserine lactone (C6-HSL), *N*-octanoyl-L-homoserine lactone (C8-HSL), *N*-(3-oxohexanoyl)-D-homoserine lactone (3-oxo-C8-HSL), and *N*-(3-hydroxydodecanoyl)-DL-homoserine lactone (3-OH-C8-HSL) were purchased from Sigma-Aldrich (St. Louis, MO, United States). 5-bromo-4-chloro-3-indolyl-β-D-galactopyranoside (X-gal) was bought from Promega (Madison, WI, United States). All other chemicals were analytical grade.

### Isolation and Identification of the Dominant Bacteria

Psychrotrophs, Enterobacteriaceae, and *Aeromonas* were identified as the dominant spoilage bacteria of vacuum packaged minced sturgeon (*Acipenser schrenckii*) stored at 4°C (Gui et al., 2014). Psychrotrophs were isolated using plate count agar (PCA, code HB0101, Qingdao Hope Biol-Technology Co., Ltd., China) after culture at 7°C for 7 days. Enterobacteriaceae were isolated using violet red bile glucose agar (VRBGA, code HB0176) after culture at 37°C for 24 h. *Aeromonas* were isolated using ampicillin macconey agar base (AMA, code HB8576) after culture at 28°C for 2 days; 32, 25, and 30 colonies of Psychrotrophs, Enterobacteriaceae, and *Aeromonas*, respectively, were selected and purified in nutrient agar plates by streaking, and then identified by 16S rDNA sequencing. For the sequencing, bacterial genomic DNA was extracted using Bacteria Genomic DNA extraction kit (Tiangen, Beijing, China). 16S rDNA was amplified by PCR using primers 27F (5′-AGAGTTTGATCCTGGCTCAG-3′) and 1495r (5′-CTACGGCTACCTTGTTACGA-3′). The PCR program was as follows: 30 cycles of denaturation at 94°C for 1 min, annealing at 52°C for 30 s, and extension at 72°C for 1.5 min, with a final elongation step at 72°C for 5 min. The PCR products were sequenced by the Beijing Biomed Biological Technology Co., Ltd. (Beijing, China). The sequences of 16S rDNA were searched against database GenBank by BLAST.

### Spoilage Ability Analysis

Twelve strains of dominant spoilage bacteria, which were list as Q1–Q4, C1–C4, and N1–N4 (**Table 1**), were inoculated into nutrient broth (at 1% inoculum size) and cultured at 30°C for 12–18 h to reach 10^9^ CFU/mL. Bacteria were collected by centrifugation at 12,000 *g*, 4°C for 10 min, resuspended in sterile saline solution, and diluted to 1.0 × 10^8^ CFU/mL; 400 mL of sterile fish muscle juice was inoculated with 20 μL of prepared bacteria and stored at 4°C to evaluate the spoilage ability of inoculated bacteria. Spoilage ability was evaluated by the microbial growth, total volatile base nitrogen (TVB-N) and sensory score. The sturgeon muscle juice was prepared according to Gu et al. (2013). TVB-N was measured as previously described (Gui et al., 2014) and expressed as g TVB-N per kg minced sturgeon muscle. Quantitative descriptive analysis (QDA) was applied to evaluate the flavor of sturgeon juice. Seven trained people were selected for the sensory panel. Surgeon juice was scored on a 0–7 point scale with 6–7 meaning fresh, 4–6 no distinct odor, 2–4 incipient rancidity, and 0–2 rancid.

**Table 1:** The bacteria isolated from vacuum packaged sturgeon stored at 4°C.

Species identification	abbreviation	Source of media	No. of isolates	Ratio of total 87 isolates (%)
*Aeromonas veronii*	Q1	AMA^a^	15	17.24
*Aeromonas media*	Q2		8	9.20
*Aeromonas sobria*	Q3		7	8.05
*Aeromonas hydrophila*	Q4		2	2.30
*Psychrobacter Cryohalolentis*	N1	PCA^b^	12	13.79
*Arthrobacter* sp	N2		4	4.60
*Pseudomonas* sp	N3		4	4.60
*Pseudomonas gessardii*	N4		4	4.60
*Citrobacter freundii*	C1	VRBGA^c^	13	14.94
*Raoultella ornithinolytica*	C2		8	9.20
*Klebsiella* spp.	C3		7	8.05
*Enterobacter* spp.	C4		2	2.30
*Serratia* spp.			1	1.15
Total			87	100

### Screening of AHL-Producing Bacteria by Biosensors

*Agrobacterium tumefaciens* KYC55 (pJZ372)(pJZ384)(pJZ410) and *Chromobacterium violaceum* CV026 were used for the detection of AHLs in “T” streak assay as previously described by Piper et al. (1993).

### Extraction of Homoserine Lactones From Culture Supernatants

*Aeromonas veronii* LP-11 was cultured in LB at 30°C, 200 rpm for 24 h. And AHLs were extracted from the supernatant of culture using method previously described (Ravn et al., 2001) with some modifications. A sample (600 mL) was centrifuged (4°C, 12,000 *g* for 10 min) and passed through 0.22 μm filter to remove cells. The supernatants were extracted three times with equal volumes of acidified ethylacetate (0.1% formic acid). The combined extract was first dried at 30°C using a rotatory evaporator .The small amount of residual liquid was dried by nitrogen gas flow. Residue was dissolved in 2 mL of HPLC-grade methanol and stored at −20°C until analysis.

### Thin Layer Chromatography (TLC) Analysis of AHLs

Acetylated homoserine lactones extract was examined by thin layer chromatography (TLC) as described by Ravn et al. (2001) with some modifications. Briefly, 2 μL of AHL extract or synthetic AHL standards was applied to a 10 cm × 10 cm sizes C18 TLC plate (TLC aluminum sheets 20 cm × 20 cm, RP-18 F254_S_, Merck 64271 Darmstadt, Germany) and developed in 60:40 (v/v) methanol/water until the front reached the top. The TLC plates were dried for at least 10 min while an agar cover layer of either *C. violaceum* CV026 or *A. tumefaciens* KYC55 was prepared. To prepare the agar top layer, 0.5 mL of preculture of *C. violaceum* CV026 or *A. tumefaciens* KYC55 was inoculated into 50 mL LB medium and grown at 30°C for 24 h with aeration. The culture was then added with 100 mL of LB-agar (1.2% agar) and immediately poured onto the TLC plates. After the agar was solidified, the TLC plates were transferred to a sealed sterile container and cultured at 30°C for 48 h. AHL standards used included C4-HSL, C6-HSL, C8-HSL, and 3-oxo-C8-HSL.

### HPLC/QqQ-MS Analysis of AHLs

HPLC/QqQ-MS analysis of AHLs extract was performed as described by Gould et al. (2006) with some modification; 10 μL of AHL extract was injected onto a C18 reverse-phase column (2.0 mm × 150.0 mm, Agilent). Mobile phase A was H_2_O containing 0.1% glacial acetic acid. Mobile phase B was methanol containing 0.1% glacial acetic acid. Elution started with 10% mobile phase B for 5 min, which then raised to 90% over 30 min and stayed at 90% for 15 min. The column was re-equilibrated for 10 min, and a blank run was performed between each analysis. The flow rate, column temperature and UV detection wavelength was 200 μL/min, 30°C, and 210 nm, respectively.

Mass spectrometric analysis was performed on Agilent 6460 triple-quadrupole tandem mass spectrometer. Precursor ion-scanning experiments were performed in positive-ion mode to monitor for *m/z* 102 in the range of *m/z* 50–400. The ion spray voltage, focusing potential, and collision energy were set at 4200, 200, and 25 V, respectively. To verify the AHLs detected in *A. veronii* extract, AHL reference standards including C6-HSL, C8-HSL, and 3-oxo-C8-HSL were analyzed in the same way.

### HPLC/QTOF-MS Analysis

10 μL sample was injected onto an Agilent SB-C18 column (2.1 mm × 50.0 mm, Agilent) and eluted at a flow rate of 400 μL/min. Mobile phase A was H_2_O containing 0.1% formic acid. Mobile phase B was acetonitrile containing 0.1% formic acid. Elution started with 10% mobile phase B for 3 min, which then raised to 100% over 21 min, stayed at 100% for 2 min, reduced to 10% over 0.1 min, and stayed at 10% for 3.9 min. The column temperature and UV detection wavelength were 30°C and 210 nm, respectively. Mass spectrometric analysis was performed on a Waters XEVO-G2 QTOF of a quadrupole and orthogonal acceleration time-of-flight tandem mass spectrometer. The scan range was *m/z* 50–600. For positive-ion mode, the capillary and cone voltage were set at 3.5 kV and 40 V, respectively. The desolvation gas was set to 600 L/h at a temperature of 450°C. The ion source temperature was set to 110°C.All data were acquired and processed using Waters MassLynxv4.1 software.

### Data Analysis

Statistical tests were performed using the SPSS 20.0 computer program (SPSS Statistical Software, Inc., Chicago, IL, United States). Data were reported as mean ± SDs of three measurements. Means were subjected to Duncan’s test and a *p*-value of <0.05 was considered significant.

## Results

### Identification of SSOs

**FIGURE 1:**
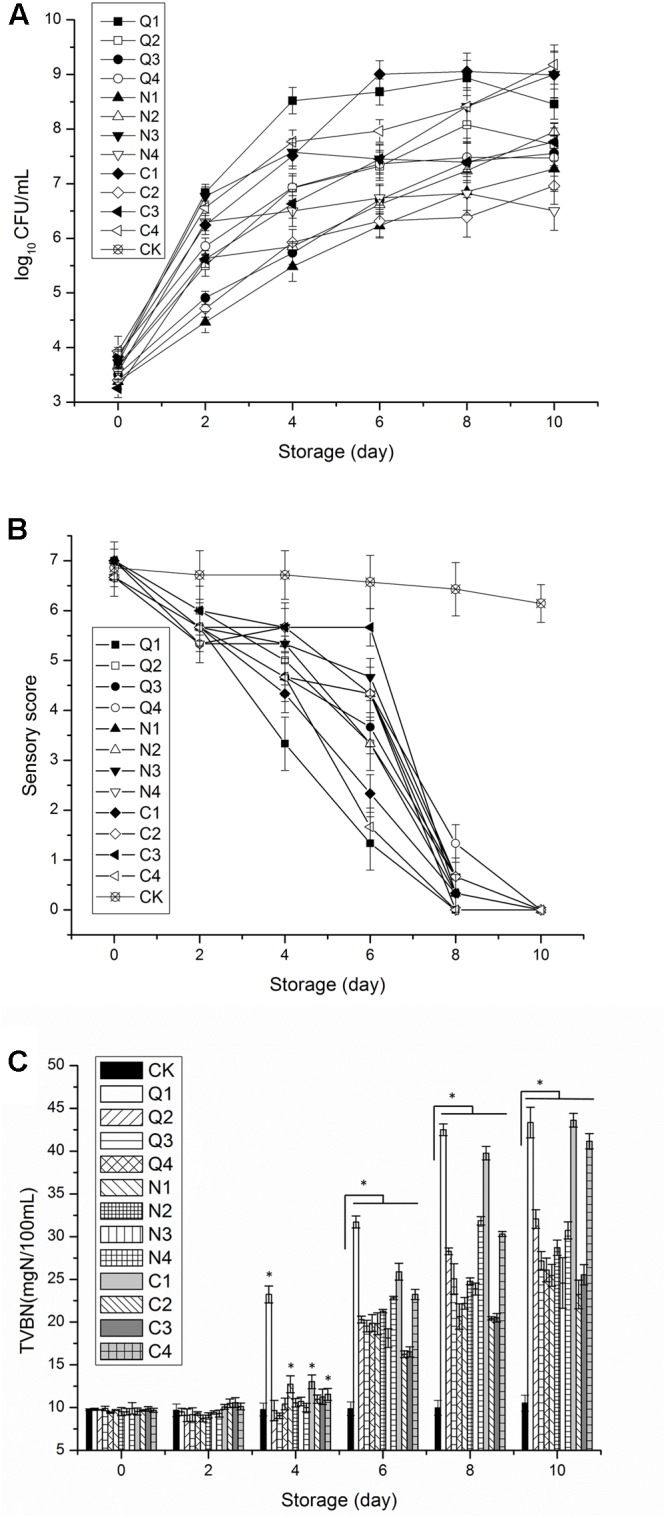
Comparison of spoilage capabilities of sterile fish juice inoculated with different bacteria (Q1–Q4, N1–N4, and C1–C4) during the storage at 4°C. **(A)** Total viable count, **(B)** sensory score, and **(C)** total volatile base nitrogen (TVB-N). The error bars indicate standard deviation (±SD). Statistical significance was marked by the asterisk (* represents *P* < 0.05).

A total of 87 strains of dominant spoilage bacteria were selected from selective mediums culture and subjected to identification by 16s rDNA sequencing. Totally, 13 species were identified from the 87 selected strains (**Table 1**). One strain from each of 12 most common identified species was selected for spoilage ability analysis. The selected 12 stains listed as Q1–Q4, C1–C4, and N1–N4 (**Table 1**) were inoculated into sterile sturgeon muscle juice. Viable counts, TVB-N values, and flavor of sturgeon muscle juice during storage were determined or evaluated. As shown in **Figure 1**, the viable count of Q1 rapidly exceeded 8 log CFU/g on day 4, which was significantly higher (*P* < 0.05) than those of all the other groups. At the end of the storage, the viable counts of Q1, N3, C1, and C4 became significantly higher (*P* < 0.05) than those of the others. The sensory scores of Q1, C1, and C4 were significantly lower (*P* < 0.05) than those of the other groups on day 6. In addition, the TVB-N values of Q1 increased rapidly after 2 days of storage and became significantly higher (*P* < 0.05) than those of all the other groups from day 4 to day8. On the final day 10 of storage, the TVB-N values of Q1, C1, and C4 increased to over 40 mg N/100 mL which were significantly higher (*P* < 0.05) than those of the other groups. Based on these data, it can be concluded that the spoilage ability of Q1, C1, and C4 was higher than that of the other tested strains. Strains Q1, C1, and C4 were classified as the SSOs of sturgeon in the present study and named as *Aeromonas veronii* LP-11, *C. freundii* LPJ-2, and *R. ornithinolytica* LPC-3, respectively.

### Biosensor Analysis

**FIGURE 2:**
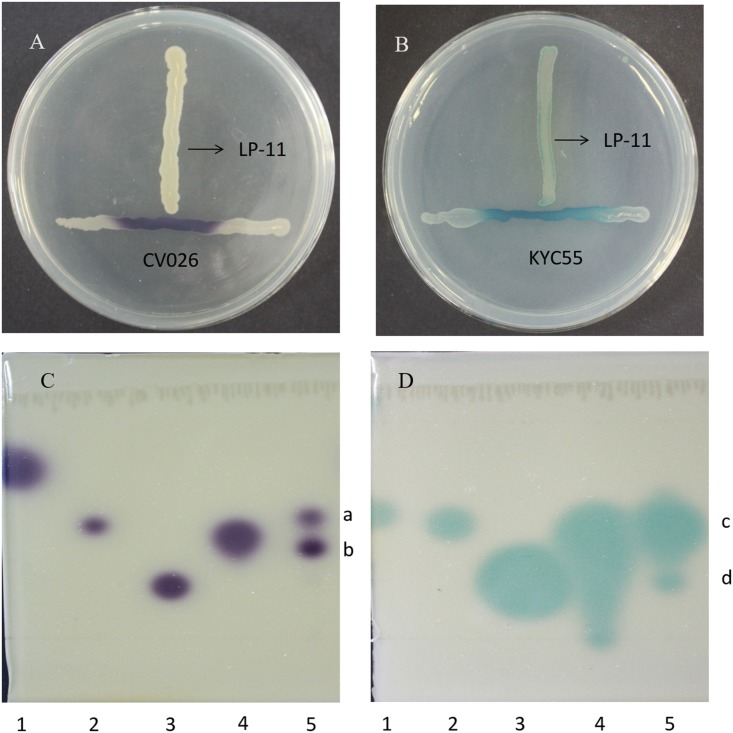
The detection of AHLs in *A. veronii* LP-11 in ‘T’ streak assay using biosensor **(A)** *A. tumefaciens* KYC55 and **(B)** *C. violaceum* CV026 and the detection of AHLs in *A. veronii* LP-11 extract by TLC using biosensor **(C)** *A. tumefaciens* KYC55 and **(D)** *C. violaceum* CV026. Lane 1, standard C4-HSL; lane 2, standard C6-HSL; lane 3, standard C8-HSL; and lane 4, standard 3-oxo-C8-HSL; and lane 5, *A. veronii* LP-11 extract.

Acetylated homoserine lactone biosensor *C. violaceum* CV026 responds well to AHLs with four to eight carbon acyl side chains but responds very poorly to AHLs with acyl chains of C10 or more (Steindler and Venturi, 2007). Another AHL biosensor *A. tumefaciens* KYC55 is sensitive to 3-oxo-substituted AHLs with acyl chain of lengths from 4 to 12 and 3-unsubstituted AHLs (Zhu et al., 2003). As each biosensor preferentially responds to AHLs with specific structure, using the two biosensors in combination can enlarge the detection range of AHLs. *A. veronii* LP-11 was able to induce violacein production in *C. violaceum* CV026 and β-galactosidase production in *A. tumefaciens* KYC55 (**Figures 2A,B**). The color of the two AHL biosensors was in gradient, with most strong response observed at the meeting point of the two strains, showing AHL production by *A. veronii*. In contrast, *C. freundii* LPJ-2 and *R. ornithinolytica* LPC-3 did not induce the QS phenomenon in the two biosensors (data was not shown). The AHLs produced by *A.veronii* LP-11 were further identified in the following study.

### TLC-Biosensor Analysis

Acetylated homoserine lactones can be partially characterized by TLC on C_18_ reversed-phase plates. Each AHL migrates with a characteristic mobility and results in a spot shape of response detected in a way depending on the reporter of the biosensor strain (Steindler and Venturi, 2007). *A. veronii* LP-11 induced two spots in the TLC plate coupled with *A. tumefaciens* KYC55 (**Figure 2C**). Spots *a* and *b* had similar mobility and shape as those of 3-oxo-C8-HSL and C8-HSL, respectively. At the same time, *A. veronii* LP-11 inducted two spots in the TLC plate coupled with *C. violaceum* CV026 (**Figure 2D**). The *R*_f_ value of spot *c* (∼0.49) was similar to that of the short chain AHL standard C6-HSL (*R*_f_∼0.49), while the *R*_f_ value of spot *d* (∼0.37) was smaller than that of 3-oxo-C8-HSL (*R*_f_∼0.41). From these results, it can be concluded that spots *a*, *b*, and *c* were 3-oxo-C8-HSL, C8-HSL and C6-HSL, respectively, but spot *d* was an unidentified AHL; these results demonstrated that at least four different AHLs were synthesized by *A. veronii* LP-11.

### HPLC/QqQ-MS Analysis

**FIGURE 3:**
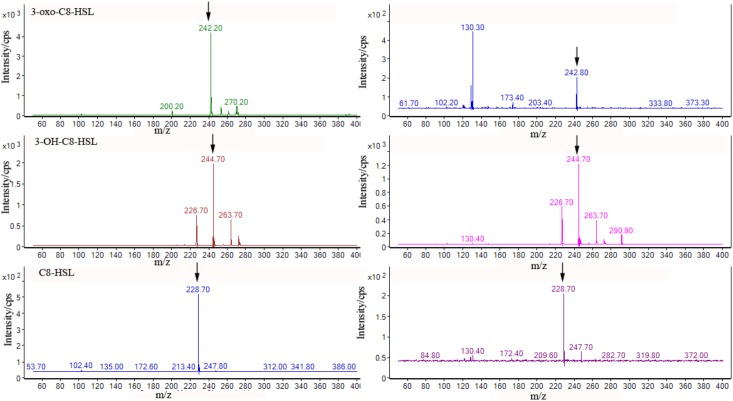
Product ion spectrum of AHLs standards **(Left)** and the corresponding peak **(Right)** in the chromatograms of *A. veronii* LP-11 extract.

To accurately identify the AHLs species synthesized by *A. veronii* LP-11, *A. veronii* LP-11 extract was analyzed by HPLC/ QqQ-MS. The ions used to identify AHLs are *m/z* 102, which corresponding to the loss of the acyl chain, and the corresponding *m/z* molecular ion (Morin et al., 2003). As shown in **Figure 3**, three AHLs were identified by HPLC/QqQ-MS. The prominent peak at *m/z* 242.8, 244.7, and 228.7 corresponded to the molecular ion [M + H]^+^ of 3-oxo-C8-HSL, 3-OH-C8-HSL, and C8-HSL, respectively. In addition, the specific retention times of molecular ions of *m/z* 244.2, 244.7, and 228.7 were in accordance with standards 3-oxo-C8-HSL (26.902 min), 3-OH-C8-HSL (27.678 min), and C8-HSL (32.932 min), respectively. Therefore, the molecular weight, retention time, and characteristic ion *m/z* 102 consistently confirmed the presence of 3-oxo-C8-HSL, 3-OH-C8-HSL, and C8-HSL in *A. veronii* LP-11 extract.

### HPLC/QTOF-MS Analysis

**FIGURE 4:**
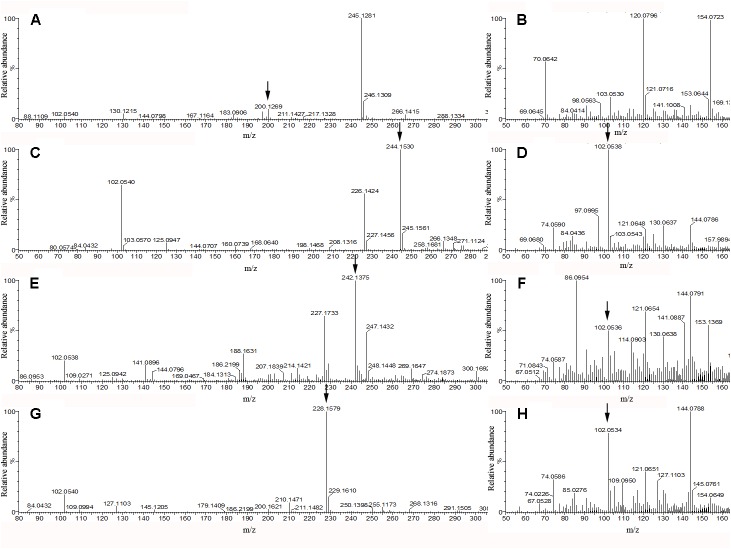
QTOF-MS spectra of four AHLs of *A. veronii* LP-11 extract. Panels **(A,B)** were the MS1 spectra and MS2 spectra, respectively of m/z 200.1269 ion; panels **(C,D)** were the MS1 spectra and MS2 spectra, respectively of m/z 244.153 ion; panels **(E,F)** were the MS1 spectra and MS2 spectra, respectively of m/z 242.1375 ion; and panels **(G,H)** were the MS1 spectra and MS2 spectra, respectively of m/z 228.1579 ion.

Thin layer chromatography analysis indicated that four different AHLs might present in *A. veronii* LP-11 extract, while QqQ-MS detected only three AHLs. To clarify the discrepancy, qTOF-MS with both high sensitivity and high mass accuracy for both precursor and product ions (Cataldi et al., 2013) was used to search the signaling molecules. The prominent precursor ions [M + H]^+^ at *m/z* 244.153, 242.1375, and 228.1579 in MS1 spectra corresponded to 3-OH-C8-HSL, 3-oxo-C8-HSL, and C8-HSL, respectively. Their intense characteristic protonated homoserine lactone product ion *m/z* 102 was observed in the MS2 spectra (**Figures 4C–F**). However, the precursor ions [M + H]^+^ at *m/z* 200.1269 which corresponded to C6-HSL were in low abundance in the MS1 spectra and no obvious characteristic product ions at *m/z* 102 were present in the MS2 spectra (**Figures 4A,B**). In order to confirm the presence of C6-HSL in the sample, standard C6-HSL was analyzed by the same method and the results showed that their retention time was the same, indicating the four compounds in **Figure 4** were 3-OH-C8-HSL, 3-oxo-C8-HSL, and C8-HSL and C6-HSL, respectively. In addition, the typical mass errors of the four compounds were below 2.1 mDa and the elementary compositions of the four compounds calculated by Marker Lynx XS software were consistent with their predicted molecular formulas (**Table 2**), which substantiated the presence of the four AHLs in *A. veronii* LP-11extract.

**Table 2:** HPLC/qTOF-MS analysis of AHLs isolated from *A. veronii* LP-11extract.

Compound	Retention time/min	Calculated formula	Mass (Da)	Calculated mass /(Da)	Error/mDa
C6-HSL	4.50	C_10_ H_18_ N O_3_	200.1269	200.1287	−1.8
3-oxo-C8-HSL	5.60	C_12_ H_22_ N O_4_	244.153	244.1549	−1.9
3-OH-C8-HSL	6.14	C_12_ H_20_ N O_4_	242.1375	242.1392	−1.7
C8-HSL	8.71	C_12_ H_22_ N O_3_	228.1579	228.16	−2.1

## Discussion

Specific spoilage organisms refer to those organisms with the highest spoilage potential in specific products or storage conditions. Identification of SSOs is important for the understanding of food spoilage process and the development of spoilage control strategies. *S. putrefaciens*, *Serratia liquefacines*, *Photobacterium phosphoreum*, *Brochothrix thermosphacta*, *Pseudomonas* spp., *Aeromonas* spp., and Enterobacteriaceae have been identified to be the SSOs in marine or fresh fish in cold storage (Gram et al., 2002; Alfaro et al., 2013). For a certain fish, SSOs of multiple or single species have been reported (Gram et al., 2002). In the present study, *A. veronii* LP-11, *Citrobater freundii* LPJ-2, and *Raoultella ornithinolytica* LPC-3 which belong to *Aeromonas* and Enterobacteriaceae were identified to be the SSOs of vacuum packaged sturgeon stored at 4°C. Enterobacteriaceae have previously been reported to be the SSO of lightly preserved fish and chilled stored meat (Gram et al., 2002). They produce indole and putrescine, leading to the spoilage of food. *Aeromonas*, mostly *A. hydrophila*, is psychrotrophic and exhibits high proteolytic and lipolytic activities in many types of food, such as fish, poultry, beef, pork, and milk (Braun and Sutherland, 2005; Ravi et al., 2008). However, *A. veronii* is seldom reported to be an SSO in food and little is known about its spoilage potential.

Specific spoilage organisms are the main cause of food spoilage. And QS was reported to be played a role in the spoilage of aquatic products (Christensen et al., 2003; Flodgaard et al., 2005; Bai and Rai, 2011; Zhu et al., 2015, 2016). In order to find out whether QS is involved in the spoilage of sturgeon, we should first confirm that whether QS signals could be produced by sturgeon SSOs. AHLs, composed of a homoserine lactone ring and *N*-acyl chains varying in length, saturation level, and oxidation state, are QS signals of a great number of gram-negative bacteria. AHLs are synthesized by the enzyme of the LuxI family and sensed by the response transcriptional regulators of LuxR family (Ng and Bassler, 2009). In the present study, the discovery of AHLs in sturgeon SSO, *A. veronii* LP-11 suggested that QS is probably associated with spoilage of sturgeon, which was demonstrated previously (Gui et al., 2017), and this result advances our understanding of the microbiology of sturgeon spoilage which may be helpful for better sturgeon preservation. In addition, the four AHLs produced by *A. veronii* LP-11 in this study were different from AHLs produced by other *Aeromonas*. In a previous study, *A. veronii* MTCC3429 was reported to produce multiple AHLs, including 6-OH-HHL, C14-HSL, and methy-branched AHLs (Thiel et al., 2009; Jangid et al., 2012). It is very interesting that a species produces so many different AHLs. The specific LuxR/LuxI system of *A. veronii* LP-11, as well as the similarity and difference in the QS system between this strain and other *Aeromonas*, needs to be further defined. Most of the reported *Aeromonas* species that produce AHLs are *A. hydrophila* and *A. salmonicida*. C4-HSL and C6-HSL are the common two AHLs produce by the two species (Bruhn et al., 2005; Medina-Martinez et al., 2006a,b; Rasch et al., 2007), while C8-HSL and 3-OH-C8-HSL, produced by *A. veronii* LP-11 in the present study, have never been identified by MS in the genus *Aeromonas* or specie *A. veronii* in other studies.

At present, there is no accepted standard method to detect AHLs from bacteria. The combined use of different AHL biosensors was a fast and convenient method to screen pure cultures of bacteria for AHL production (Steindler et al., 2007). Using biosensors, we screened the three SSOs in sturgeon and only *A. veronii* LP-11 induced both the two biosensors (**Table 1**), indicating that it produced AHLs. *C. freundii* LPJ-2 and *R. ornithinolytica* LPC-3 were not found to produce AHLs by the two biosensors. This could be explained by two reasons: (i) the lack of a functional luxI homolog gene for production of AHLs; (ii) the amount of AHLs produced by these two strains was below the detection threshold of the biosensors (Chang et al., 2012). In addition, the deficiency of lasI homolog gene or the growth medium used in the study could be the reason for the low production of AHLs either (Zhu et al., 2002, 2004). Four AHLs including C4-HSL, C6-HSL, C8-HSL, and 3-oxo-C8-HSL, were identified in *A. veronii* LP-11 extract by qTOF MS. With this result, it can be determined that spots *c* and d in TLC plates coupled with *A. tumefaciens* KYC55 were 3-oxo-C8-HSL and C8-HSL, respectively, and spots *a* in TLC plates coupled with *C. violaceum* CV026 were C6-HSL. As 3-oxo- and 3-hydroxy derivatives of AHLs of the same chain length migrate with indistinguishable mobility in the methanol/water solvent system (Shaw et al., 1997), it can be deduced that spot *b* was 3-OH-C8-SHL. *A. tumefaciens* KYC55 has been reported to have the greatest sensitivity to AHLs and the broadest AHL range (Zhu et al., 2003), but it still failed to detect C6-HSL and 3-OH-C8-HSL in the present study. Interestingly, all the four AHLs were detected when *A. tumefaciens* KYC55 and *C. violaceum* CV026 used in combination, suggesting that the combined use of the two biosensers is necessary. QqQ-MS is an effective and sensitive approach for analysis of AHLs. It has been used to detect AHLs in *Tenacibaculum maritimum*, *Escherichia coli*, and *P. aeruginosa* extract (Gould et al., 2006; Romero et al., 2010). However, it was failed to detect C6-HSL in the present study. This may contributed by two reasons: (i) the concentration of C6-HSL in *A. veronii* LP-11 extract was too low to meet the detection limit of QqQ-MS and (ii) the interference of other compounds in the *A. veronii* LP-11 extract which had the same retention time of C6-HSL, produced too many fragment ions, and reduced the intensity of the target ion in the mass spectrum. These may also explain why C6-HSL showed low intensity of precursor ions [M + H]^+^ in the MS1 spectra and no obvious characteristic ion *m/z* 102 in the MS2 spectra in TOF MS. In contrast, the four AHLs were all accurately identified by qTOF-MS with typical mass errors below 2.1 mDa within 30 min, indicating that qTOF-MS was more effective and accurate than the methods of TLC and QqQ-MS.

## Conclusion

The study demonstrated that *A. veronii* LP-11, *C. freundii* LPJ-2, and *R. ornithinolytica* LPC-3 were the SSOs of vacuum packaged sturgeon stored at 4°C. Among the three SSO, only *A. veronii* LP-11 produced C6-HSL, C8-HSL, 3-oxo-C8-HSL, and 3-OH-C8-HSL. So far as we know, this is the first report that C8-HSL and 3-OH-C8-HSL were detected by MS in the genus *Aeromonas*. Our results showed that HPLC/qTOF-MS was the most versatile detection method for rapid analysis of AHLs in complex microbial sample. This is the first attempt to characterize AHLs produced by sturgeon spoilage bacteria. Further studies are needed to clarify the QS regulation mechanisms of the four AHLs and the role of AHLs in sturgeon spoilage.

## Author Contributions

MG and PL designed the study and drafted the manuscript. RW and LL collected test data and interpreted the results. SW and JH provided useful suggestions of the study.

## Conflict of Interest Statement

The authors declare that the research was conducted in the absence of any commercial or financial relationships that could be construed as a potential conflict of interest.
